# Adsorption Performance Analysis of Alternative Reactive Media for Remediation of Aquifers Affected by Heavy Metal Contamination

**DOI:** 10.3390/ijerph15050980

**Published:** 2018-05-14

**Authors:** Antonio Molinari, Celia Margarita Mayacela Rojas, Amerigo Beneduci, Adalgisa Tavolaro, Maria Fernanda Rivera Velasquez, Carmine Fallico

**Affiliations:** 1 Dipartimento di Ingegneria Civile, Università della Calabria, 87036 Rende (CS), Italy; margaritamayacela@hotmail.com (C.M.M.R.); carmine.fallico@unical.it (C.F.); 2 Dipartimento di Chimica e Tecnologie Chimiche, Università della Calabria, 87036 Rende (CS), Italy; amerigo.beneduci@unical.it; 3 Consiglio Nazionale delle Ricerche, Istituto per la Tecnologia delle Membrane, CNR-ITM, Università della Calabria, 87036 Rende (CS), Italy; a.tavolaro@itm.cnr.it; 4 Faculty of Engineering, National University of Chimborazo, Riobamba EC060104, Ecuador; mafer.rivera@live.com

**Keywords:** contaminants dynamics, batch test, zeolite, natural fibers, zero valent iron, heavy metals in groundwater

## Abstract

A series of experimental batch tests has been carried out with the aim of improving the knowledge of fundamental processes related to the fate and behavior of heavy metals that can be of environmental concern in groundwater. The analysis of contaminants (i.e., Cu, Zn, Cd and Pb) dynamics in different environmental compartments is specifically addressed by comparing the removal efficiencies of different types of reactive materials, three natural (i.e., vegetal fibers, natural limestone and natural zeolite) and one synthetic (i.e., synthetic zeolite). Results stemming from these reactive media has been compared with the outcomes related to the same test performed using zero valent iron which is the reactant usually employed for heavy metals remediation. All tested reactants exhibited important removal percentages, even larger than 90% in most cases, achieved in a contact time ranging between about 12 h and slightly longer than a day (i.e., 30 h). Maximum adsorption percentages are observed for pH ranging between 4 and 8 for all tested materials and contaminants. Our findings provided relevant evidence, to both researchers and technicians, on the competitiveness of the explored alternative mediums with respect to the classical reactants usually employed for heavy metals remediation.

## 1. Introduction

Groundwater represent about 96% of the freshwater reserves of the planet [1] and its pollution can constitute a serious risk to human health [2]. In this framework, groundwater remediation is a fundamental tool to ensure environmental safeguards and acceptable levels of quality. Useful technologies for remediation of contaminated aquifers are, for instance, pump and treat or permeable reactive barriers (PRB) [3,4,5,6,7,8,9]. Although widespread, the pump and treat methodology is often inadequate to achieve target concentrations since its effectiveness depends strongly on the hydrogeology of the site. On the other hand, PRB allows in situ degradation of contaminants forcing the polluted plume to pass through a reactive medium placed in a dedicated trench. The proper choice of the reactive material is fundamental for the achievement of the remediation goals [10]. For the treatment of heavy metals, the reactivity of many substances was investigated in the past considering different processes such as precipitation, adsorption, ion exchange, and reverse osmosis [11,12,13,14,15,16,17,18]. Nevertheless, these methods present several disadvantages, such as the high cost and the limited availability of reactive materials. For instance, good reactants usually employed are the activated carbons which are well known for their effectiveness in removing both organic and inorganic substances, such as heavy metals [11]. Chen and Wang [12] obtained important result for Cu, Zn and Pb removal of from waste water using a fixed bed of granular activated carbon pretreated by deionized water at a given pH. The appropriate choice of the reactive material for a PRB depends mainly on the contaminant type and other factors such as the hydrogeological conditions of the aquifer or the environmental impact associated with the pollutant to be removed. Obiri-Nyarko et al. [19] reported an overview of the most used materials for PRB and treated contaminants. Currently, with particular reference to heavy metals contamination, the reactive medium usually employed for real-scale applications in PRB is zero valent iron (ZVI), which is able to reduce oxidized organic species, chlorinated solvents, heavy metals and radionuclides [20,21,22,23,24]. However, the effectiveness of ZVI is often questionable. In fact, the percolation of pollutant solutions in soil was considered, for a long time, to be a potential cause of groundwater quality deterioration. Moreover, the reactivity of the iron as a reducing agent can be influenced significantly by various substances occurring within the system, such as organic material or metal oxides which, reacting with iron, tend to form complexes giving rise to precipitates. The latter can in turn lead to the presence of impurities within the dedicated trench producing alterations in the value of porosity and hydraulic conductivity of the system, which can affect the whole effectiveness of the PRB [25]. Other reactive materials employed for heavy metals removal through a PRB system are limestone, TRM (transformed red mud), zeolites, and peat moss. Limestone requires careful control of the pH and tend to form precipitates that can clog the barrier and reduce its hydraulic performances. In general, calcareous materials allow important removal percentages to be obtained, especially when are combined with inert materials and sands to improve the permeability of the barrier and sometimes with the addition of organic matter (i.e., compost) to stimulate microbial activity. For instance, by using this mix a reduction of Pb equal to 100% was obtained [26]. Zeolites have been demonstrated to be very effective for Pb, Cu and Cd removal with percentages up to 95% due to their high cation-exchange capacity and their insensitivity to pH changes [13,14,27]. TRM, namely brine mixed with waste materials (red mud), has an alkaline behavior in nature (pH of 8–10.5) and shows a good removal efficiency for Fe, Cu, Zn, Ni, and Pb [28]. The application of the peat within a PRB allows significant removal percentages to be obtained due to its large specific surface area (>200 m^2^/g) and high porosity (95%). Moreover, it exhibits good ion exchange properties leading to important removal percentages (30–65%), similar to those of zeolites, and high removal capacity of Cd, Cr, Ni, and Zn. In the literature are several studies concerning the removal efficiencies of alternative mediums such as carbon nanotubes [29], formaldehyde resin [30], and magnetic carbon spheres derived from Fe(III) [31]. Several types of contaminants can be treated by selecting appropriate reagents [32], and for this reason the investigation of new reactive media more economically convenient and devoid of the drawbacks found for ZVI represents a continuous challenge.

In the present study, we explored the removal efficiencies of four reactive materials with respect to four heavy metals, copper (Cu), zinc (Zn), cadmium (Cd) and lead (Pb), which can occur in contaminated groundwater or in wastewater. The reactive materials investigated are three natural reactants (a vegetal fiber, a natural limestone and a natural zeolite) and a synthetic zeolite. The results obtained from the investigated media were compared with those stemming from the same test performed using ZVI.

The overall results of our work concern a first phase of investigation targeted at the comprehension of the reactivity of these substances in order to provide relevant evidence on the competitiveness of these alternative mediums with respect to the classical reactants usually employed for heavy metals remediation.

## 2. Materials and Methods

Within this study we basically considered five reactive materials, namely three natural, from Ecuador, and two commercially available synthetic.

### 2.1. Natural Materials Tested

#### 2.1.1. Cabuya Fibers

**Figure 1:**
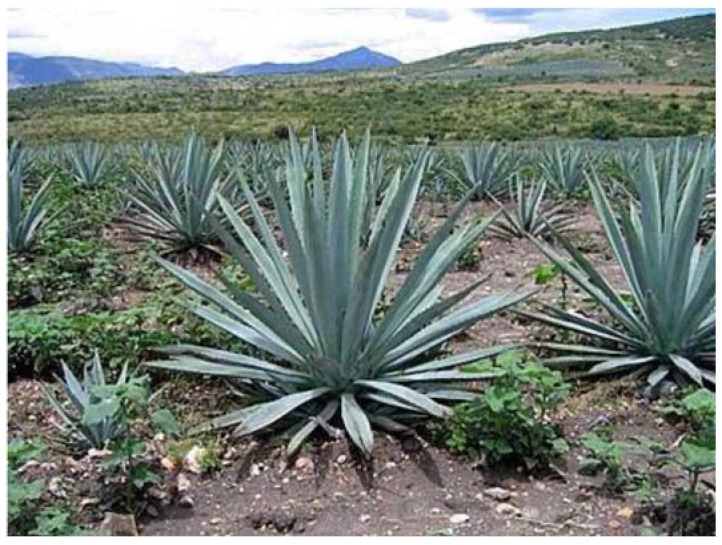
Cabuya or maguey (scientific name: *Furcraea andina*).

Cabuya, also known as fique fiber, is a natural fiber extracted from the *Agave* cactus plant, belonging to the *Furcraea microphylla* species which present similar characteristics to the sisal fibers [33]. *Agaves* are essentially plants typical of the tropical areas and their characteristic genus consists of up to nearly 300 species, all of which are native of tropical and sub-tropical North and South America, but many of which have been introduced in other countries such as East Africa, West Indies, Indonesia, South Africa, Philippines, etc. Fibers from *Agave* plans (Figure 1) are obtained from the structural fiber which is contained in the fleshy leaves of the plant.

There are several varieties of *Agave* plants and among these we focused on *Furcraea Andina* type. The fiber is extracted from the leaves by hand, or by means of small machines using a technique called decortication. With extraction by hand, one man can extract about 4.5 kg of fiber per day and the yield of the fiber is between 3% and 4% of the weight of the leaf, whereas if machines are used the yield is much lower, namely up to 2%. Most of the fiber produced is used for the manufacture of rice bags and other uses are for making twine, rope, etc. [34].

Looking at technical properties for engineering applications, Navacerrada et al. [35] collected several properties of the fique fibers (i.e., cabuya) from different research studies obtaining the following main values:

- Water absorption: 60.0% as average value [33];
- Tensile strength (MPa): 43–571 [33] or 132–262 [36];
- Elongation (breaking) (%): 6–9.8 [33] or 4.8–10.6 [36];
- Elasticity modulus (GPa): 8.2–9.1 [33] or 3.9–7.5 [36].

Due to these characteristics cabuya fibers are usually employed to produce bio-composite fibro-reinforced materials (e.g., [33,37]) and several studies are available in literature, while very little attention has been devoted to the investigation of this natural material for remediation purposes.

#### 2.1.2. Ecuador Limestone

Ecuador limestone (termed also as *piedra caliza*) employed for our investigation is basically a carbonate mineral naturally occurring worldwide. In specific, the limestone used for our experiments was collected in Ecuador, Riobamba. Limestone is a sedimentary rock mostly composed by calcite mineral with varying crystal forms of calcium carbonate (CaCO_3_) [38]. Various researchers reported the large potential of limestone in removing several heavy metals such as Cd, Pb, Zn, Ni, Cu, Fe, Mn and Cr from landfill leachate (e.g., [39,40]), Mn from water and wastewater, Fe from groundwater [41] and for the treatment of acid mine drainage (e.g., [42,43]).

#### 2.1.3. Natural Zeolite

The natural zeolite used for our experiments was collected in Ecuador, Riobamba. In general, natural zeolites are basically formed from basaltic lava in specific rocks that are subjected to moderate geologic temperature and pressure. Depending on the formation environment, natural zeolites can have different chemical composition and cation-exchange capacity. From a geochemical point of view, natural zeolites are hydrated aluminosilicate minerals with a porous structure and several peculiar physicochemical properties, such as cation exchange, molecular sieving, catalysis and sorption. The aluminosilicate constitute the primary building block of the natural zeolites framework and, depending on atoms arrangement at the vertexes, these can be identified with different structural types. Water molecules can be present in voids of large cavities and can form bonds between internal ions and exchangeable ions via aqueous bridges.

Due to its structure, an important property of zeolites is the ability of ions exchange with the external medium. This behaviour of a natural zeolite depends on several factors such as the global structure, surface charge density, ion size and shape, ionic charge and concentration of the external solution [43,44]. On the other hand, adsorption performances of natural zeolites are influenced by Si/Al ratio, cation type, number and location of adsorption sites [45]. Due to these properties, in past decades natural zeolites have found a variety of applications in adsorption, catalysis, building industry, agriculture, soil remediation (e.g., heavy metal removal), and energy [44].

### 2.2. Synthetic Materials Tested

#### 2.2.1. Synthetic Zeolite

For our study, zeolite 4A (molecular sieve 4A) powder was purchased from Aldrich Chemicals (8–12 mesh, CAS Number 70955-01-0|Linear Formula Na_12_[(AlO_2_)_12_(SiO_2_)_12_]·*x*H_2_O). Natural water of hydration is removed from this network by heating to produce uniform cavities which selectively adsorb molecules of a specific size leading to different types of applications such as ion exchange, molecular sieves and catalysis. Depending on vertex-sharing of SiO_4_ and AlO_4_ tetrahedra, about 100 different architectures of synthetic zeolites are known. On the basis of pores diameter, different types of zeolites can be identified. In this study, we considered the A-type zeolite which is characterized by pore diameters between 3 Å (angstrom) and 10 Å (1 nm). A 4- to 8-mesh sieve is normally used in gasphase applications, while the 8 to 12-mesh type is common in liquid phase applications. In our experiments, we employed the zeolite 4A which has a pore diameter of 0.36–0.40 nm (around 4 Å). The layout structure of zeolite 4A is schematically shown in Figure S1 of the Supplementary Material. The negatively charged framework of zeolite 4A is balanced by the presence of cations such as Na^+^ or Ca^2+^. Two different chemical formula representations of zeolite 4A are widely used, Na_12_Al_12_Si_12_O_48_ has a pseudocell with unit cell parameter of 12.292 Å, while the true ideal unit cell has the chemical formula Na_96_Al_96_Si_96_O_384_ with a unit cell parameter of 24.555 Å.

#### 2.2.2. Zero Valent Iron (ZVI) 

For our tests, we employed iron powder, type FG 1000/2000/ZVI, grain size 1–2 mm commercialized by iPutec GmbH & Co. KG (Rheinfelden, Switzerland). Nowadays, ZVI represents the most commonly employed reactive reagent for heavy metals remediation through a PRB technology. In recent decades, ZVI has gained increasing attention for the remediation/treatment of both groundwater and wastewater contaminated by both organic and inorganic compounds such as organic chlorates, nitroaromatic compounds, dyes and phenol, various heavy metals, nitrates [46], radionuclides [47] etc. Several studies have demonstrated the good ability of ZVI for removing different types for metallic ions such as Cr(VI) [48,49], Ni^2+^ [50], Pb^2+^ [51], Cu^2+^ [52], and Zn^2+^ [53].

The loss of dissolved metal concentrations from the solution usually corresponds to a direct increase in pH caused by the anaerobic dissolution of ZVI [54] which allows contaminated slurries to be treated through a combination of redox processes (some of which may be biologically mediated, such as the reduction of sulfate), precipitation reactions and sorption, all of which are surface-mediated and require contact between a reactive surface site and the contaminant.

### 2.3. Heavy Metals Investigated

Four heavy metals, specifically Cu, Zn, Cd and Pb, were selected for this study. In the following, we report a brief description of the speciation, chemistry and environmental behaviour of these contaminants.

Because of the higher frequency of the presence of nitrate ions in soils, we used the nitrates of these metals to prepare our contaminated slurries: cadmium nitrate tetra-hydrate [Cd(NO_3_)_2_·4H_2_O], lead nitrate [Pb(NO_3_)_2_], zinc nitrate hexa-hydrate [Zn(NO_3_)_2_·6H_2_O], copper nitrate tri-hydrate [Cu(NO_3_)_2_·3H_2_O]. For these contaminants, we considered concentrations larger, from 10 to about 40 times, than the current regulatory limits in Italy in order to simulate the occurrence of a strong polluted slurry which needs to be remediated. Specifically, we carried out our experiments considering the following initial concentrations: Cu 43,000 μg/L (regulation limit = 1000 μg/L), Zn 65,339 μg/L (regulation limit = 3000 μg/L), Cd 50 μg/L (regulation limit = 5 μg/L) and Pb 177 μg/L (regulation limit = 10 μg/L). These concentrations are compatible, for instance, with mine drainage pollution which has been demonstrated to cause severe impacts on biological systems resulting in a strong contamination of water resources and related environments over many decades to thousands of years [55,56].

### 2.4. Laboratory Investigations

Experiments were performed employing a batch setup where tested materials are placed in a confined vessel together with a contaminated solution of the single heavy metal considered, assuming the same initial concentration. To explore the behavior of each tested material we considered different contact times. Batch tests were carried out with each metal separately with the goal to identify the efficiency of each filter material for removal of each tested metal. The aim was the analysis of the removal efficiencies, namely the concentration variations in solution over time, of each material tested with respect to the four contaminants considered within this study, and to derive relevant aspects to employ these alternative materials for remediation purposes.

For each test we employed 12 independent batch reactors with the same amount of tested material (i.e., 300 mg) and of contaminated solution (i.e., 3 mL) where each reactive medium has been tested against 12 different contact times. The initial pH of the metal cation solutions was between 5 and 6. To explore removal efficiencies over time, we considered the following contact times during which each reactor has been constantly agitated: 0.5, 1, 2, 4, 6, 8, 10, 14, 22, 24, 26, 30 h. Depending on the heavy metal tested, we considered a solution with a concentration larger than 10 to about 40 times the current regulation limits in Italy. When the fixed contact time has been reached the batch reactor, namely the test tube, was centrifuged for 10 min at 2000 rpm and then the water sample was passed through a 0.45 μm hydrophilic syringe filter (Minisart, Goettingen, Germany) to analyze the soluble fraction and then acidified with HNO_3_ (pH = 2) before analysis. Dissolved heavy metals concentration has been then quantified by means of an ICP-MS (inductively coupled plasma mass spectrometry) iCapQ Thermofischer. The instrument was calibrated using different analytical standards concentrations (Fluka TraceCERT^®^ multielement standard solution for ICP, certified reference materials (CRM)) in the range of 0.1–50 ppb. All chemicals and reagents used for the experiments had an analytical grade of purity. Ultrapure water (18.3 MΩ cm, Arioso-Human Corporation, Seoul, Korea) was used for all the solutions.

### 2.5. Characterization of the Investigated Materials

#### 2.5.1. Cabuya Fibers

Previous studies (e.g., [57,58,59,60]) showed that the lignin content in a natural fiber can play an important role in the process of adsorption of metal cations. On the other hand the adsorption may depend also on other factors such as metal concentration in solution, amount of reactive fibers, or the contact time between solution and fiber. Some authors [33,61,62] found the following main composition: cellulose 63–80%, lignin 10.1–17%. In comparison with Spanish broom (*Spartium* *junceum*), which exhibits 6.6% of lignin content [63] and for which Fallico et al. [59,64] and Arias et al. [60] highlighted significant adsorption capacity for heavy metals, we observed a larger lignin content in cabuya fibers. Depending on the fiber type, namely row or elementary (i.e., subjected to further extraction procedure from the row fibers), different measurements are available in literature (e.g., [65,66,67,68]).

We analyzed the structure of the cabuya fibers taking several scanning electron microscope (SEM) pictures (instrument brand: FEI Company; type: Quanta Inspect 200) both in a longitudinal and a cross section view (Figure S2 of the Supplementary Material) at different magnifications. As observed by Kozlowski et al. [65] and Delvasto et al. [33], we measured a diameter of 274.71 μm (Figure S2b of the Supplementary Material). From the cross-sectionional view we observed a characteristic net structure (Figure S2c,d of the Supplementary Material) similar to the cross section of other natural fibers, observed from different SEM images, and presented by other authors (e.g., [66]). We also characterized cabuya fibers by means of X-ray diffraction (XRD) powder analysis (Figure S3 of the Supplementary Material). The analysis evidences an XRD pattern characterized by the occurrence of diffraction peaks of cellulose (C_6_H_10_O_5_)_n_, of the type I_β_ [69], and lignin. Cellulose diffraction peaks can be observed in correspondence with the two major peaks approximately centered at 2-theta, respectively, of about 18° and 26°. Literature analysis highlighted that, depending on the fiber type (i.e., hardwoods or softwoods) and the lignin type analyzed, the average peak associated with the occurrence of lignin can be found within a 2-theta range of 19.3–22.7° [70,71,72].

#### 2.5.2. Ecuador Limestone

The mineralogical composition of the limestone was analysed by XRD and ICP-MS elemental analysis after complete digestion (i.e., mineralization) in the laboratory. Mineralization was performed by acid attack with a 3/1 (*v*/*v*) HNO_3_/HF solution and microwave digestion at 120 °C for 1 h (Milestone Start D digestion system). The significant peak observed at 2-theta of about 30° (Figure S4 of the Supplementary Material) indicated that the limestone used in our study is mainly composed of calcite (CaCO_3_), as also found by Allende et al. [73] who observed a similar pattern. Moreover, the second relevant peak observed at 2-theta of about 27° was consistent with the occurrence of quartz (SiO_2_). XRD powder analysis revealed that Ecuador limestone is composed of a mixture of well-crystallized concrete and quartz materials. SEM microphotographs (Figure S5 of the Supplementary Material) confirm the presence of a homogeneous material formed by inter-growth crystalline clusters and highlight (i) the typical porous structure of the material which results in high porosity and large adsorption surface, and (ii) the occurrence of several aggregated flakes. Elemental analysis by ICP-MS after sample mineralization, as described before, highlighted the fact that Ecuador limestone is mainly composed by Na, K and Fe while Al, Ti and Si occur in lower amounts. Other elements are present at trace level or absent.

#### 2.5.3. Natural Zeolite

The mineralogical composition of the natural zeolite used for our experiments was analysed by XRD while its elemental composition by ICP-MS after complete digestion (i.e., mineralization as described before) was undertaken in the laboratory. The significant peaks observed within the 2-theta range of about 26–29° (Figure S6 of the Supplementary Material) indicate that the natural zeolite employed in our study is basically composed by mordenite ((Ca, Na_2_, K_2_)Al_2_Si_10_O_24_·7H_2_O) which is one of the most abundant zeolites occurring in altered volcanic deposits and commonly occurs as white, glassy needles filling veins and cavities in igneous rocks. The highest peak observed at 2-theta of about 27° is consistent with the occurrence of quartz (SiO_2_). The XRD powder pattern of natural zeolite reveals a crystalline structure consisting of a mixture of zeolite, a mordenite framework, and of quartz. SEM observations (Figure S7 of the Supplementary Material) seem to confirm the presence of a crystalline material and reveal that the natural zeolite is characterized by (i) a typical surface structure which lead to large specific surface and high porosity, and (ii) the occurrence of clusters and granules. ICP-MS elemental analysis of the mineralized natural zeolite (data not shown in this paper) highlighted that our zeolite sample is mainly composed by Na, K, Si and Fe (the latter occur with the largest content) while Al and Ti occur with lower amounts. Other elements are present at trace level or absent.

#### 2.5.4. Synthetic Zeolite

We characterized the powder of the synthetic Zeolite 4A by SEM analysis (Figure S8 of the Supplementary Material) selecting groups of aggregates exhibiting smaller optical reflection. SEM pictures revealed: (i) the typical spherical shape of aggregates groups consistent with the occurrence of small crystals constituting the synthetic zeolite employed for our tests with an aspect ratio (length to the width) equal to about 1; and (ii) the large porosity which characterize this material.

#### 2.5.5. ZVI

The surface properties of the ZVI employed in our tests have been characterized by SEM (Figure S9 of the Supplementary Material) which revealed the occurrence of stripes that can be viewed as the occurrence of large specific adsorption surfaces.

## 3. Results and Discussion

In the following sections, we present the main outcomes stemming from the batch tests performed. For each of the four heavy metals considered, we discuss the adsorption trends observed during our experiments for each reactive material investigated.

### 3.1. Adsorption Trends Analysis

#### 3.1.1. Copper

**Figure 2:**
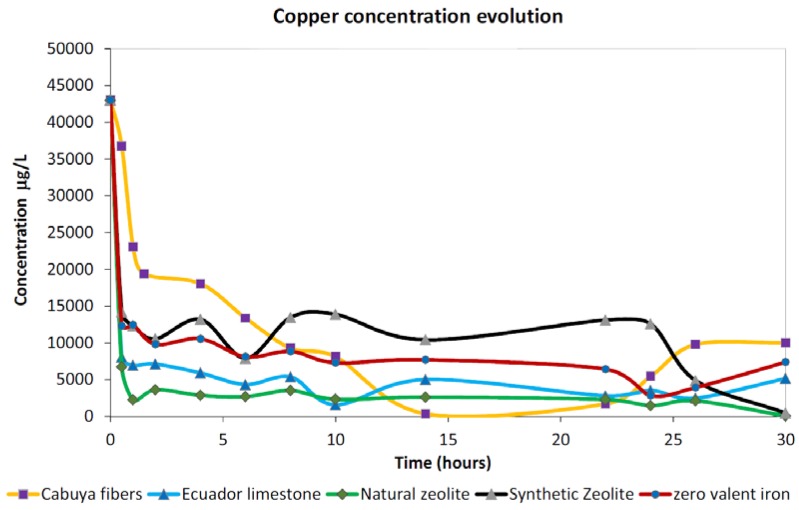
Observed trends for copper investigations for the five tested materials.

Figure 2 reports observed adsorption trends in the case of a copper-contaminated solution for the five reactive materials considered within this study. We considered, for each material, the same initial concentration of 43,000 μg/L, basically 43 times the Italian regulation limit for copper (1000 μg/L). Considering the same contact times (i.e., 30 h) for all investigated materials, we observed similar decreasing trends but with different performances in term of amount and rate of copper removal from the same initial solution. After 2 h of contact time all materials exhibited a sharp decrease in dissolved copper concentration from the initial value, while in the rest of the test the decrease was less pronounced.

Comparison among the observed trends for the five tested materials highlighted that in the case of a copper-contaminated slurry, the best adsorption performance was exhibited by the natural zeolite which observed the lowest dissolved values during the whole test, even if comparable concentrations were also detected in the case of Ecuador limestone. On the other hand, the lower ability of copper removal during the test was observed in the case of the synthetic zeolite which displayed an important adsorption behavior only at the end of the test highlighting a different adsorption kinetic than the other materials investigated.

The best adsorption performance was observed in the case of cabuya fibers, which showed the maximum removal percentage at about the halfway point of the test (i.e., 14 h of contact) while other materials displayed similar percentages at the end of the test (see Table 1). The fastest adsorption kinetic was exhibited by the natural zeolite which, after 2 h of contact time, largely adsorbed dissolved Cu which observed a steep decrease in solution. By contrast with the other materials, in the case of cabuya fibers, an increasing trend in dissolved concentrations seemed to occur after about 22 h of contact time. Despite the fact that this could suggest a possible releasing effect of Cu^2+^ ions from the fibers, the observed increase in dissolved concentrations was related to a short observation period which did not allow us to identify a clear releasing effect resulting from a possible specific modification of the adsorption properties of this material after a certain contact time. In fact, due to the limited duration of the tests, it cannot be excluded that the increase in the concentration of copper was due to specific episodes, probably not definitive, which can be attributed to different causes, such as, for example, a temporary decrease in the specific surface of the fiber and of the associated functional groups caused by the alteration of these, localized manifestations of spontaneous compaction of fibrous material, or even changes and instability of the solution pH in the final part of the test. To ensure that the investigated materials reached the saturation of the pollutant, further investigations are needed employing a larger duration than those adopted for our tests.

#### 3.1.2. Zinc

**Figure 3:**
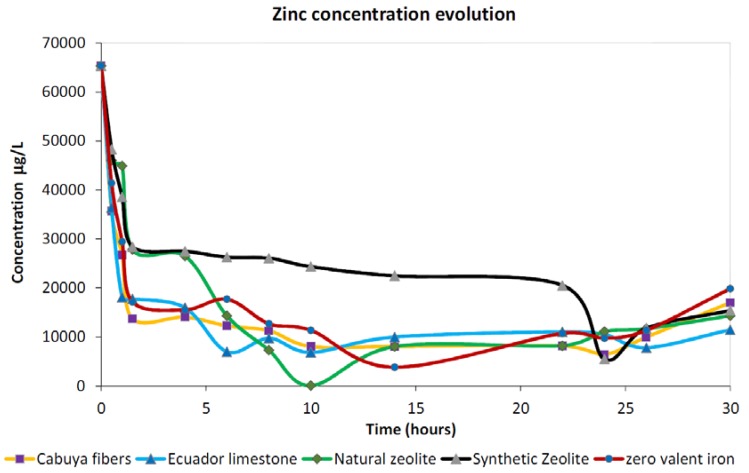
Observed trends for zinc investigations for the five tested materials.

Figure 3 depicts the comparison between the adsorption trends observed in the case of zinc for the five tested materials. As well as observed in the case of copper, for zinc also after about 2 h of contact between the reactive medium and the contaminated solution for all investigated materials a sharp decrease in dissolved concentrations was observed, consistent with the occurrence of a large number of available adsorption sites. Analyzing the trends for long durations it is quite clear that cabuya, limestone and ZVI behaved in a similar manner exhibiting similar final concentrations. The worst adsorption performances were displayed by the synthetic zeolite which, after the initial 2 h of sharp decrease, exhibited a very slow linear decreasing trend up to 22 h of contact time after which a new sharp decrease was observed and then followed by an increase in dissolved concentrations for the next 6 h. This latter behavior can be seen as a releasing trend but it is important to consider that this oscillation in concentration values was observed over a short period and hence did not allow us to identify a clear releasing trend. In any case, what was specified above for copper applies also for zinc i.e., to exclude the influence of variations in the concentration due to various situations, localized over time, a longer time range for the tests should be employed in future investigations. On the other hand, natural zeolite showed the best adsorption ability since it is the only material which, during the test, attained concentrations very close to zero. The good properties of the natural zeolite with respect to a zinc-contaminated solution are highlighted by (a) the limited time required to reach the maximum adsorption point than the other tested materials; and (b) the low detected concentrations after the maximum adsorption point. In fact, this natural zeolite is able to remove basically almost all the zinc occurring in the initial solution after about 10 h of contact. Moreover, after 10 h of contact its release was practically comparable to the maximum adsorbed concentrations of the other tested materials which displayed similar trends up to the end of the test.

#### 3.1.3. Cadmium

**Figure 4:**
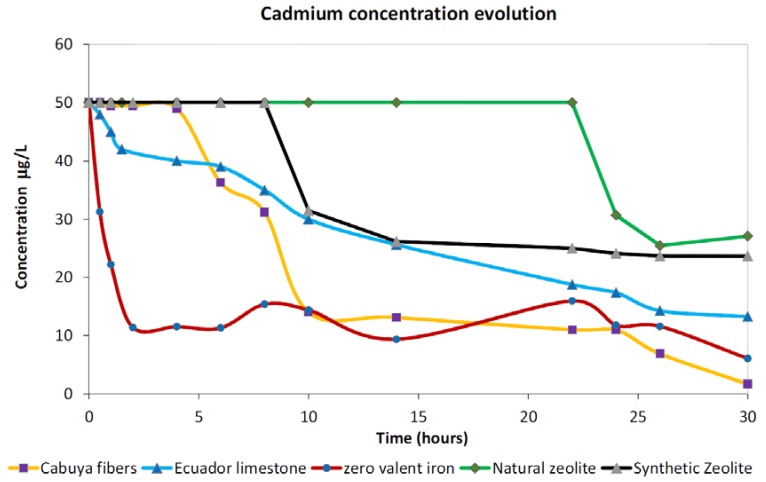
Observed trends for cadmium investigations for the five tested materials.

Very different adsorption trends were observed in the case of cadmium whose resulting concentrations during the test are reported in Figure 4. Trend analysis highlights the low ability of both zeolites, namely the synthetic and the natural, to adsorb cadmium from the solution for the duration employed during our investigations consistent with the possible low affinity of these materials with this cation. Deep analysis of the observed adsorption trends revealed that, despite both zeolites exhibiting similar final concentrations at the end of the test, natural zeolite begins cadmium adsorption only after about 22 h of contact time, highlighting the occurrence of a longer adsorption kinetic for this material with respect to this contaminant in comparison with the corresponding synthetic material, which exhibited an initial adsorption time closer to cabuya fibers and other investigated materials.

Good adsorption properties were observed for the limestone, which showed two linear decreasing trends, one more pronounced from the beginning of the test to about 2 h of contact time and the other, less steep, from 2 h to the end of the test. This observation highlights the possibility that this material can continue to adsorb cadmium for a duration longer than 30 h.

In the case of the cabuya fibers, no adsorption was observed for about the first 5 h of the test while the reduction of dissolved concentrations started with a steep slope up to 10 h of contact time. After this duration, we observed a pseudo constant behavior for about 12 h (from about 10 to about 22 h of contact time) which was followed by a new decrease in dissolved concentrations, suggesting the possibility of reaching zero dissolved value for durations larger than 30 h of contact time.

Completely different behavior than the other explored materials was observed for the ZVI, which exhibited a sharp decrease in the first 2 h of contact time and then showed a constant trend with fluctuations of about 12 μg/L for about 22 h (from about 4 to about 26 h from the beginning of the test). At the end of the test we observed a new relevant decrease in dissolved concentrations, which suggests the beginning of a new adsorption kinetic able to further reduce cadmium concentrations for durations longer than 30 h.

#### 3.1.4. Lead

**Figure 5:**
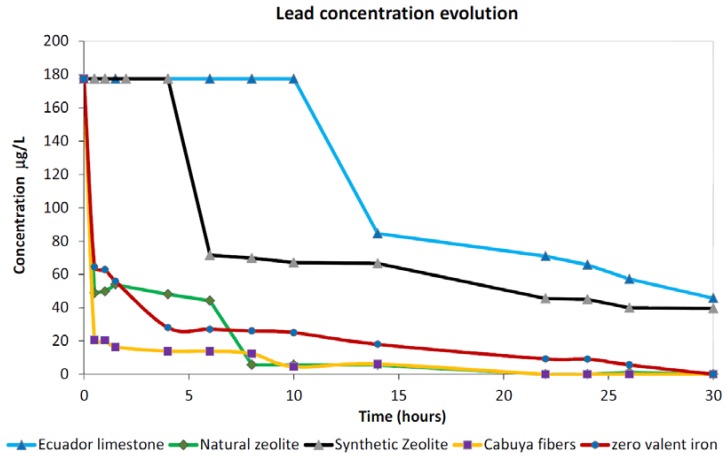
Observed trends for lead investigations for the five tested materials.

Observed adsorption trends for lead are reported in Figure 5 which highlights three different behaviors, with the synthetic zeolite exhibiting an intermediate performance between natural zeolite and the other three tested materials. The different observed behaviors can be related to the different needs of each material to be preventively hydrated in order to allow adsorption activation with respect to this metal. Cabuya fibers, ZVI and limestone displayed similar trends with a relevant sharp decrease within the first hour of contact time followed by a slow decreasing trend up to the complete adsorption of the metal at the end of the test. On the other hand, limestone started to adsorb the metal only after more than 10 h of contact time with two distinct linear trends, the first sharper than the second, which did not achieve the complete removal of the dissolved lead, suggesting the need for a longer contact time than that explored during our test in order to remove a larger amount of this metal from the solution. An intermediate behavior was exhibited by the synthetic zeolite which, after 4 h of contact time, displayed an abrupt decrease in dissolved concentrations similar to that observed for the ZVI in the first hour of contact time. After that, the adsorption trend followed a linear decrease with a similar slope than that observed for the other materials.

Looking at the natural zeolite, we noted that after the sharp decrease of the first hour it exhibited a pseudo constant trend for about four hours (i.e., from about 2 to 6 h) followed by a new relevant decrease at about 7 h of contact time to attain values close to zero, which kept up to the end of the test. Similar adsorption kinetics were observed for cabuya fibers and ZVI which, from about 4 h of contact time up to the end of the test, displayed a linear decreasing trend with a similar slope.

Excluding limestone and synthetic zeolite, all the tested materials adsorbed all the dissolved lead occurring in solution within the duration explored for our test highlighting the potential large efficiency of these materials in lead remediation.

### 3.2. Heavy Metals Removal Comparison

The overall comparison of adsorption performances for the five tested materials with respect to each heavy metal investigated within our study is reported in Table 1, where removal percentages and contact times refer to the maximum adsorption point.

Analyzing the adsorption performances in the case of copper investigations, we noted that all tested materials were very efficient in removing this metal from the contaminated slurry examined since in all cases we obtained removal percentages larger than 90%. Looking at contact times corresponding to the maximum adsorption percentage, we observed that cabuya fibers exhibited a very fast kinetic with respect to other materials and more specifically to the synthetic zeolite, which reached comparable dissolved concentrations only at the end of the test after 30 h of contact. On the other hand, natural zeolite demonstrated the ability to adsorb, after 30 h of contact, basically all copper occurring in the initial slurry, exhibiting a removal percentage very close to 100%. The worst adsorption performance, even if larger than 90%, was observed in the case of the ZVI which is usually employed for slurry remediation.

With respect to zinc we noted that all reactants were very efficient in removing this metal, exhibiting a maximum adsorption percentage very close to or larger than 90%. In specific, the best adsorption performance was attained by the natural zeolite which in about 10 h adsorbed basically all the dissolved zinc, while the others showed similar removal percentages within the range 89–94%. The slowest adsorption performances were exhibited by cabuya fibers and synthetic zeolite which reached their maximum removal percentages in 24 h while the other investigated materials showed a maximum adsorption point after a shorter contact time.

Looking at the adsorption study for cadmium in correspondence of the maximum adsorption point, we observed significant different behaviors for the investigated materials. In fact, important removal percentages were observed in the case of cabuya fibers, Ecuador limestone and ZVI which displayed values larger than 70%. On the other hand, the two zeolites (natural and synthetic) exhibited about 50% of removal, highlighting a possible low affinity of these materials with cadmium. All tested reactants showed a long adsorption kinetic for cadmium in comparison with other metals considered in this study as highlighted by the observation that all maximum adsorption points were attained by the end of the test.

Regarding lead adsorption performances, we observed that all tested materials offered important adsorption rates exhibiting removal percentages larger than 70%. In specific, cabuya fibers, natural zeolite and ZVI displayed a total removal of dissolved Pb concentrations, while Ecuador limestone and synthetic zeolite showed significant adsorption properties of around 75% of removal. For all tested reactants, the maximum adsorption performances were attained by the end of the test after 22 or 30 h of contact time, depending on the tested material, highlighting the need for long contact times to ensure good remediation levels with respect to this metal.

### 3.3. Analysis of the Main Removal Mechanisms

To analyze the adsorption performances of a given reactive material with respect to dissolved heavy metals in depth, it is important to consider the proper determination of the so called point of zero charge (PZC) which is defined as the pH at which a given adsorption surface has a net charge equal to zero, namely an overall neutral charge [74]. In fact, the same medium (i.e., the same reactive surface) can adsorb cations or anions depending on the pH of the solution with respect to the typical PZC. As a result, for pH values lower than the PZC the surface can be positively charged and hence may adsorb anions, while for pH values larger than the PZC the same reactive surface can be negatively charged and thus may adsorb cations. On these bases, we determined the PZC for the five reactive media used in our study. The analysis of these values, listed in Table 2, highlights that cabuya fibers and natural zeolite basically demonstrate the same PZC and can exhibit a neutral behavior at pH close to 7 while Ecuador limestone and ZVI, which also show basically the same PZC, could not exhibit adsorption abilities for pH values close to 8. The absence of adsorption can be observed for synthetic zeolite in the presence of alkaline conditions at pH of about 9.6.

Analyzing the mechanisms which led to the different removal percentages observed in the case of each reactive material involved in our study, we observed that the relevant mechanism responsible for the large adsorption percentages observed is related to the ability of the natural fibers to fix dissolved metals as a result of the binding process enhanced by the lignin and cellulose content, which are able to affect metal speciation and sorption as also observed by other authors (e.g., [58,75,76]). In fact, properties such as the relatively low molecular weight, non-solubility in water, and high specific surface reveal the good adsorption capacities of lignin for metals, as also stated by other studies (e.g., [57,76,77]). The overall results stemming from our batch tests (see Table 1) proved that cabuya fibers were very effective for the removal of all heavy metals tested with the following order of removal efficiencies Pb > Cu > Cd > Zn.

In the case of Ecuador limestone, heavy metal removal could basically be attributed to the combination of different effects. One mechanism can be related to the rough surface of the limestone which offers solid contact for metal ions promoting the sorption on available sites, consistent with the findings of Reddy et al. [78] in the case of calcite. Another process is linked to the internal structure of the limestone composed of calcium carbonate which, once dissolved in solution, can cause an increase in pH producing metal precipitation as oxides, as also stated by others (e.g., [38]). An important role is also played by the ion exchange with calcium, which can lead to the formation of metal carbonated compounds. In fact, limestone is basically a carbonate mineral which can lead to a high level of metal removal due to the formation of metal carbonate or metal hydroxide chemical precipitates [38]. Regarding Cd and Zn cations, some studies [79,80] showed that they are strongly sorbed by the calcium carbonate surface. The results of our batch tests highlighted the fact that Ecuador limestone was very effective for Cu and Zn removal while moderate removal efficiency was found for Cd and Pb. According to our findings (Table 1), the removal efficiencies during our experiments increased in the following order: Cu > Zn > Pb > Cd, as also found by Reddy et al. [78] in the case of calcite, with the exception of Pb, which in our case exhibited a lower performance than that observed by Reddy et al. [78].

Heavy metal removal in the case of both natural and synthetic zeolite can be attributed to processes such as ion exchange, precipitation, electrostatic adsorption of metal cations to the negatively charged sites occurring on zeolite particle surfaces, and surface complexation mechanisms. In fact, the negatively charged surface of these materials is balanced by exchangeable cations, such as Na, K or Ca, which can be exchanged with other cations, such as Cu, Zn, Cd and Pb, which can occur in a contaminated aqueous solution as found in other studies (e.g., [81,82,83,84]). Excluding Pb, both zeolites observed very close maximum removal percentages highlighting the fact that both follow similar adsorption mechanisms. With the exception of Cd, for all tested metals natural zeolite showed larger adsorption percentages than the synthetic zeolite. In fact, the increase in alkalinity can promote metal sorption via surface complexation processes due to the large specific surface of natural zeolite as also recognized by others (e.g., [85]).

On the basis of our results (Table 1), the removal efficiencies in the case of the natural zeolite are basically the same for Cu, Zn and Pb, while Cd exhibits about the half of the removal performances observed for the other three metals. In the case of the synthetic zeolite, the adsorption efficiencies during our experiments increased in the following order: Cu > Zn > Pb > Cd, as also observed in the case of Ecuador limestone.

ZVI removes dissolved heavy metals by several mechanisms such as reductive transformation, ion exchange and adsorption/co-precipitation processes. ZVI has strong reducing capacities favoring electrochemical reduction of dissolved metals [24] which, as a result of iron corrosion in water, can precipitate in the form of metal hydroxide and hydroxide complexes when the concentration of iron hydroxides in solution increase causing a pH rise. In fact, due to ZVI oxidation, an excess of available electrons is released in solution and tends to pass to contaminants which undergo a reductive mechanism resulting in precipitation or degradation. Another recognized mechanism is the replacement of dissolved metals with iron ions in the form of iron oxide or hydroxide [86]. Considering our results, we observed the following increasing adsorption efficiencies during our experiments: Pb > Zn > Cu > Cd.

## 4. Conclusions

Our findings confirm that the natural materials tested can provide competitive adsorption results in relation to the more expensive artificial reactants usually employed for heavy metal removal. In specific, we derived the following major conclusions:

- Large adsorption percentages (>90%) can be observed by employing, for the remediation of Cu- and Zn-contaminated slurry, all materials considered in the present study. Important removal performances can be observed after about 12 h of contact time in batch conditions. Cd removal by employing reactants whose main mechanism is surface adsorption, such as those considered in the present study, can require up to 25 h or more of contact time, in batch conditions, before useful removal percentages are observed, especially in the case of zeolites, both natural and synthetic, which can result in quite ineffective performances for the adsorption of this metal. High-adsorption performances for Pb can be observed for ZVI, cabuya fibers and natural zeolite, while results that are not competitive can be observed in the cases of limestone and synthetic zeolite.
- Cabuya fibers and natural zeolite can exhibit a neutral adsorption behavior at pH close to 7, while Ecuador limestone and ZVI could not exhibit adsorption abilities for pH values close to 8. The absence of adsorption can be observed for synthetic zeolite in alkaline conditions (pH = 9.6).
- Due to field conditions where several factors (e.g., rainwater recharge of groundwater reservoirs, redox and pH changes, organic matter occurrence, flux conditions, water-table oscillations and so on) can play a key role in the dynamics of dissolved species, our findings, obtained under controlled conditions, cannot be directly transferred to field applications. At the same time, our study demonstrates that all tested materials, both natural and synthetic, can be very effective at heavy metals removal, exhibiting a large potential for adsorption of these types of contaminant. Removal percentages stemming from our findings observed comparable and sometimes larger values than ZVI, which is the reactant usually employed for PRB remediation, highlighting the fact that these alternative media, after further field test investigations, can be considered as very competitive for the treatment of either groundwater or wastewater.

## Figures and Tables

**Table 1:** Removal performances for the five tested materials with respect to each heavy metal considered.

		Tested Materials				
		Cabuya Fibers	Ecuador Limestone	Natural Zeolite	Zero Valent Iron (ZVI)	Synthetic Zeolite
Cu	Removal %	99.24	96.33	99.89	93.42	98.98
	Contact time (hours)	14	10	24	24	30
	Optimum pH	5.75	6.18	6.58	5.04	7
Zn	Removal %	90.14	89.52	99.9	94.17	91.65
	Contact time (hours)	24	10	10	14	24
	Optimum pH	6.85	6.67	6.81	5.87	7.19
Cd	Removal %	96.67	73.43	49.09	87.83	52.66
	Contact time (hours)	30	30	26	30	30
	Optimum pH	7.26	7.46	7.75	4.87	8.01
Pb	Removal %	100	74.23	100	100	77.67
	Contact time (hours)	22	30	22	30	30
	Optimum pH	6.45	7.04	6.78	4.06	8.16

**Table 2:** Point of zero charge (PZC) values for reactive media used in our study.

Tested Material	pH
Cabuya fibers	6.79
Natural zeolite	6.81
ZVI	8.00
Ecuador limestone	8.10
Synthetic zeolite	9.57

## Supplementary Materials

The following are available online at http://www.mdpi.com/1660-4601/15/5/980/s1, Figure S1: Schematic view of the structure of zeolite 4A, Figure S2: Scanning electron microscope (SEM) pictures of cabuya fibers in (**a**), (**b**) longitudinal view and in (**c**), (**d**) cross-sectional view with different zooms, Figure S3: X-ray diffraction (XRD) pattern of cabuya fibers, Figure S4: XRD pattern of limestone, Figure S5: SEM pictures of Ecuador limestone with (**a**) 200x and (**b**) 6000x magnifications, Figure S6: XRD pattern of natural zeolite, Figure S7: SEM picture of natural zeolite, Figure S8: SEM pictures of (**a**) a particle and (**b**) specific zoom on its surface of the synthetic zeolite, Figure S9: SEM pictures of zero valent iron (ZVI) with two different magnifications.
