# Supplementary material for: Adsorption Performance Analysis of Alternative Reactive Media for Remediation of Aquifers Affected by Heavy Metal Contamination
Source: Int J Environ Res Public Health. 2018 May 14;15(5):980. doi: 10.3390/ijerph15050980 (PMC5982019; doi:10.3390/ijerph15050980)
Supplement: Supplementary file 1 [file ijerph-15-00980-s001.pdf]

International Journal of
Environmental Research and Public Health

MDPI

Supplementary Materials: Adsorption Performance Analysis of Alternative Reactive Media for Remediation of Aquifers Affected by Heavy Metal Contamination

Antonio Molinari [1,*], Celia Margarita Mayacela Rojas [1], Amerigo Beneduci [2], Adalgisa Tavolaro [3], Maria Fernanda Rivera Velasquez [4] and Carmine Fallico [1]

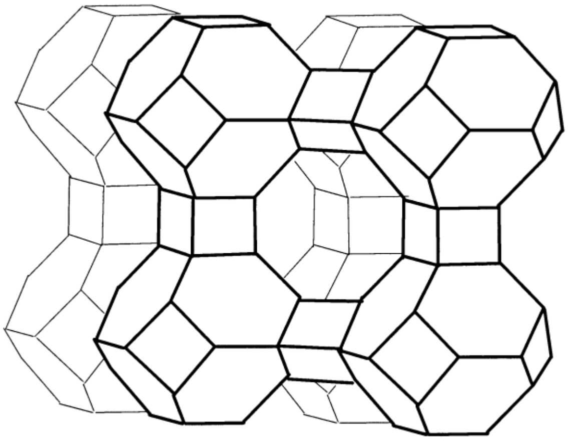

Figure S1. Schematic view of the structure of zeolite 4A.

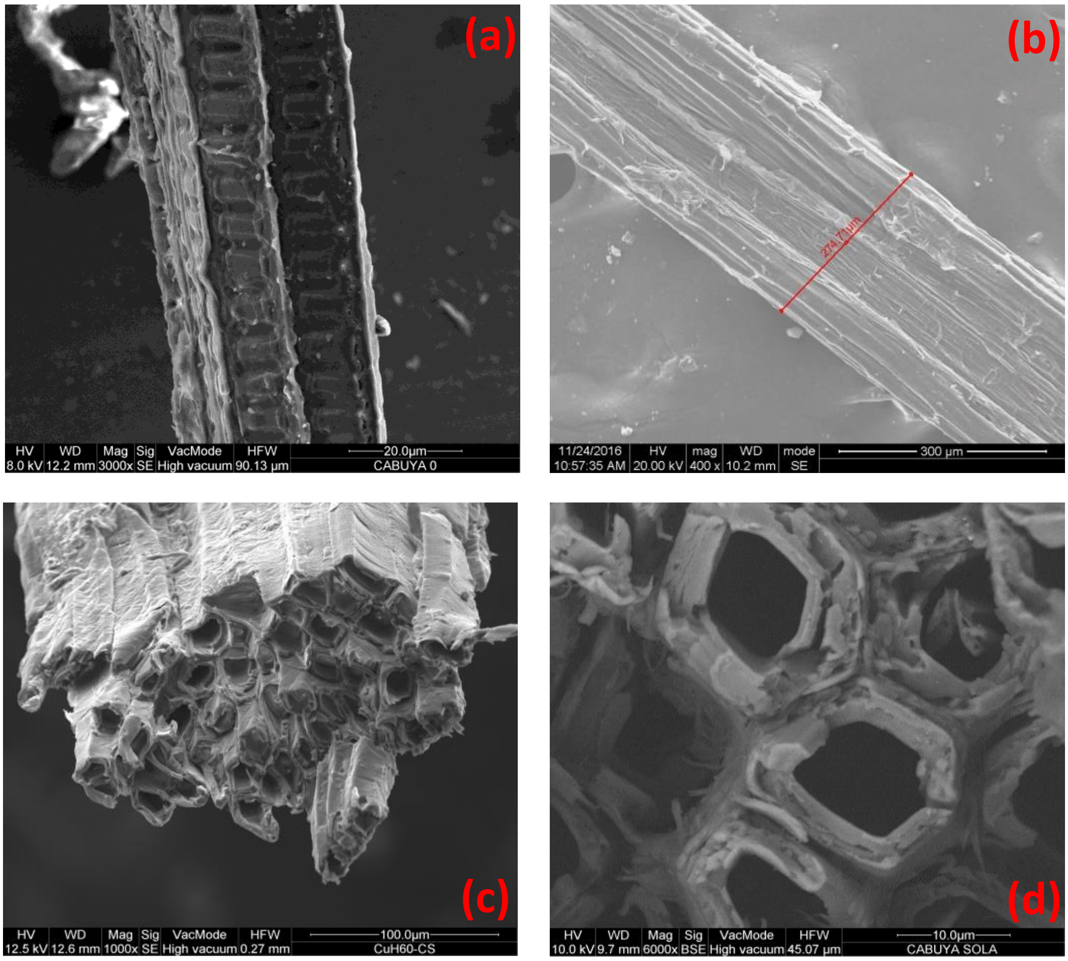

Figure S2. Scanning electron microscope (SEM) pictures of cabuya fibers in (**a**), (**b**) longitudinal view and in (**c**), (**d**) cross-sectional view with different zooms.

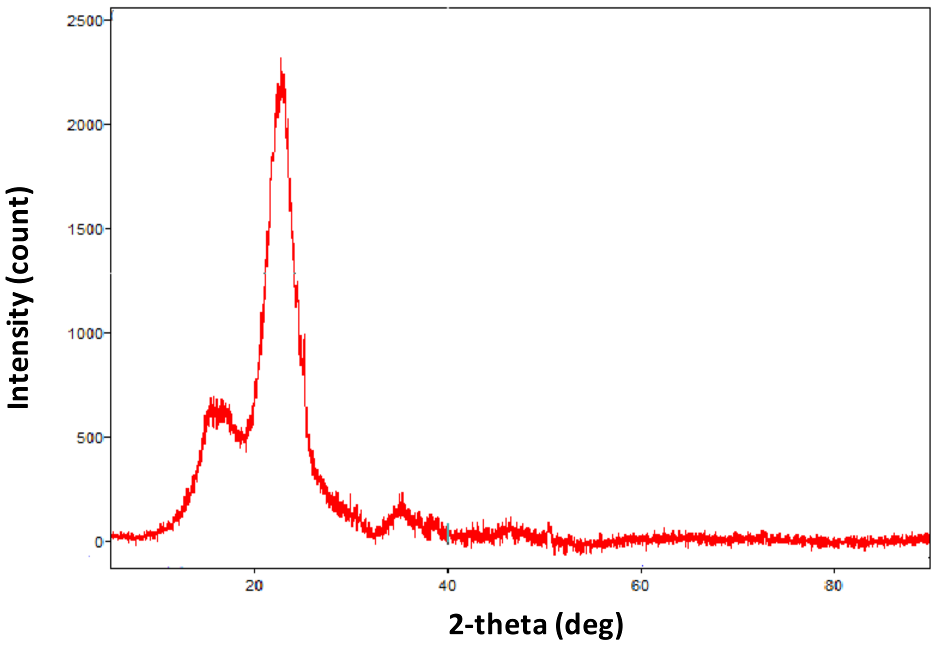

Figure S3. X-ray diffraction (XRD) pattern of cabuya fibers.

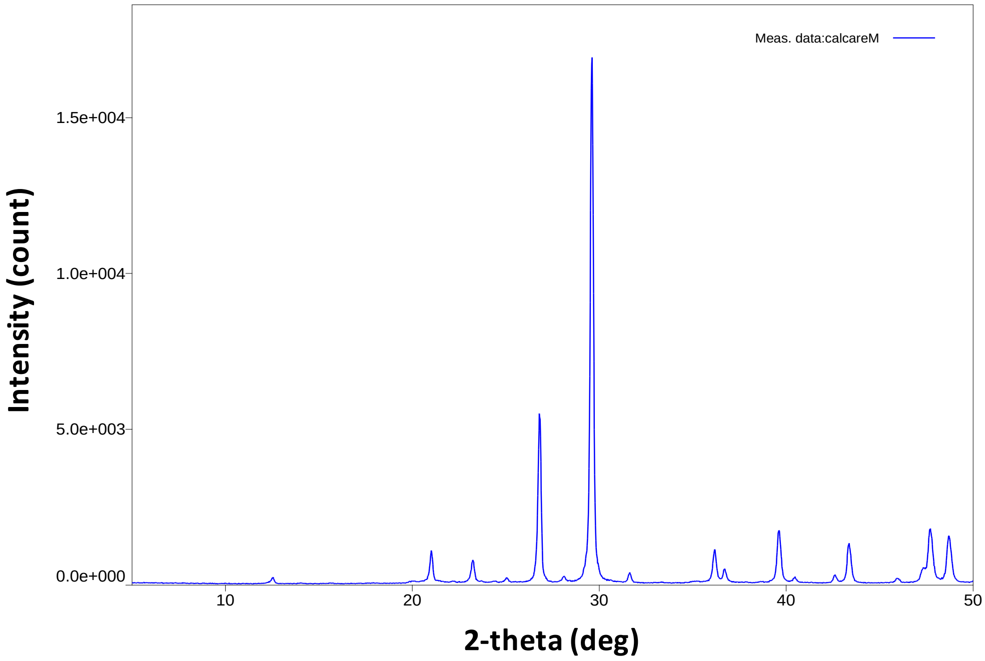

Figure S4. XRD pattern of limestone.

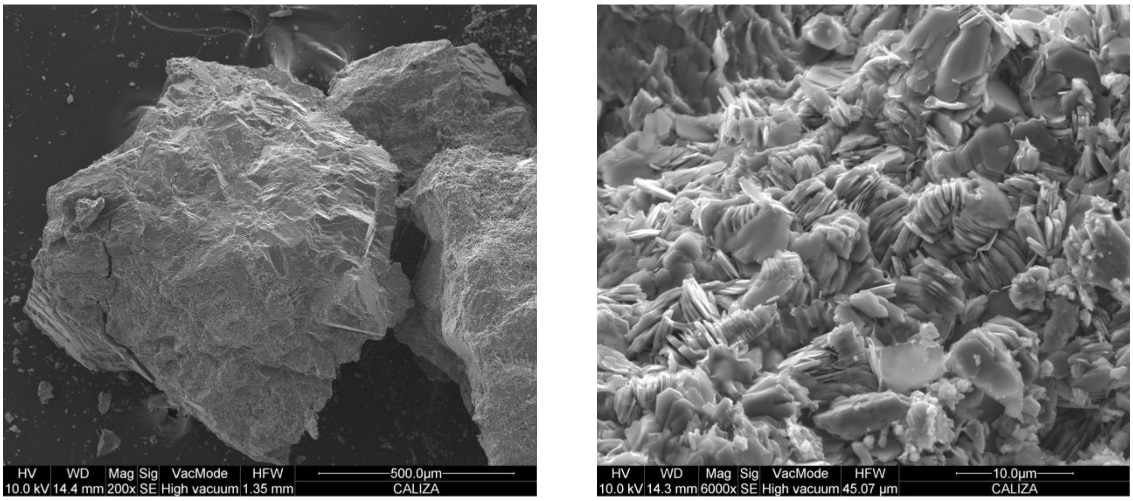

Figure S5. SEM pictures of Ecuador limestone with (a) 200x and (b) 6000x magnifications.

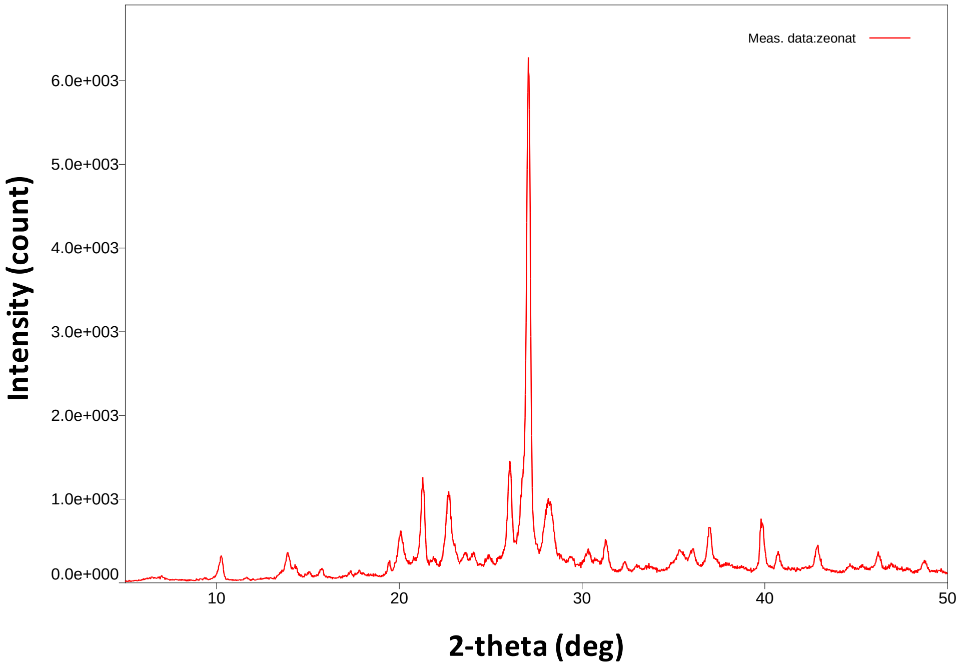

Figure S6. XRD pattern of natural zeolite.

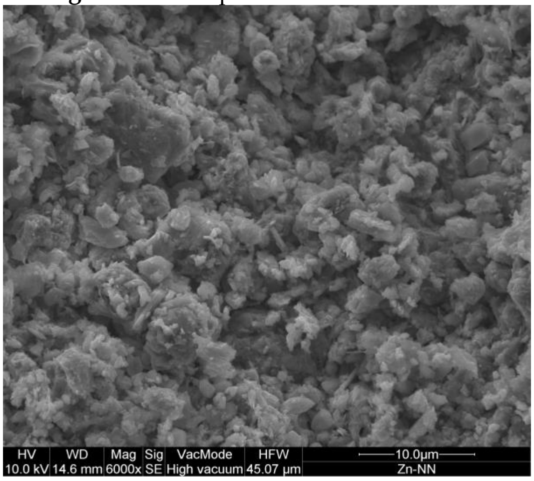

Figure S7. SEM picture of natural zeolite.

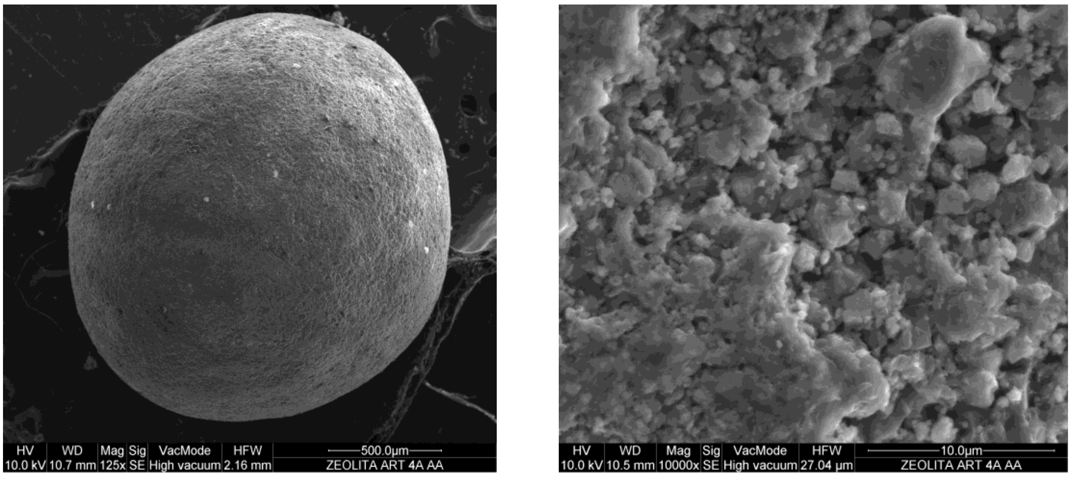

Figure 8S. SEM pictures of (a) a particle and (b) specific zoom on its surface of the synthetic zeolite**.**

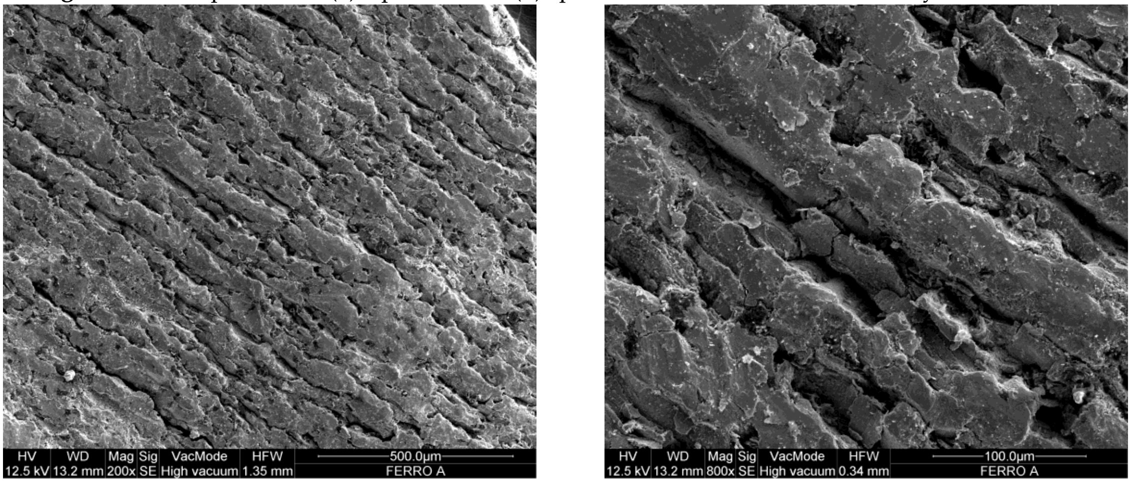

Figure 9S. SEM pictures of zero valent iron (ZVI) with two different magnifications.

© 2017 by the authors. Submitted for possible open access publication under the terms and conditions of the Creative Commons Attribution (CC BY) license (http://creativecommons.org/licenses/by/4.0/).
